# P056: The patient experience of the mrsa screening process and the impact of a MRSA positive result: a qualitative study

**DOI:** 10.1186/2047-2994-2-S1-P56

**Published:** 2013-06-20

**Authors:** H Loveday, A Tingle, K Currie, C Lafarge, J Prieto, O Freeman, A Whitfield

**Affiliations:** 1 University of West London, London, UK; 2 Glasgow Caledonian University, Glasgow, UK; 3 University of Southampton, Southampton, UK

## Introduction

Universal screening for methicillin resistant Staphylococcus aureus (MRSA) of admissions to hospital became mandatory in England in 2010 . However, there is little data about the patient experience of MRSA screening, the impact of receiving a positive result and the confidence patients have in the care they receive.

## Objectives

To explore MRSA screening from the perspective of the patient, assess its role in maintaining confidence in efforts to prevent healthcare associated infection, and create patient reported experience measures (PREMs) to inform the future development of screening policies.

## Methods

Semi-structured telephone interviews were conducted with patients who had a recent experience of MRSA screening in three National Health Service trusts. Patients found to be MRSA positive were asked about their experience of decolonisation treatment. Transcripts from digital audio files of the interviews were entered into NVivo software and underwent content analysis using a deductive approach.

## Results

Interviews were conducted with 23 patients. In general, MRSA screening was accepted as part of the hospital routine and contributed to reassuring participants about hospitals’ commitment and ability to prevent infection. Participants recommended that more information about the screening procedure should be provided, particularly the results of the screen, even if negative. Reactions to being MRSA positive varied from initial shock, a sense of being embarrassed or stigmatized, concern over being a danger to others, to frustration with recurrent colonisation. While decolonisation at home presented few problems, there was evidence of incorrect use of products.

## Conclusion

To secure and sustain patients’ satisfaction and confidence in procedures to prevent MRSA infection patients should be provided with individualised information, both written and verbal. Staff should be sufficiently knowledgeable and confident to invite questions and communicate information in a sensitive way. Specific guidelines for home-based decolonisation are required. The PREMs generated by this study are an essential tool to enable services to measure and act on feedback from the patient experience.

## Disclosure of interest

None declared

